# Inhibitory effect of kaolin minerals compound against hepatitis C virus in Huh-7 cell lines

**DOI:** 10.1186/1756-0500-7-247

**Published:** 2014-04-17

**Authors:** Liaqat Ali, Muhammad Idrees, Muhammad Ali, Abrar Hussain, Irshad Ur Rehman, Amjad Ali, Syed Abbas Iqbal, Eyad Hassan Kamel

**Affiliations:** 1 Division of Molecular Virology, National Centre of Excellence in Molecular Biology, University of the Punjab, Lahore, 87-West Canal bank Road, Thoker Niaz baig, Lahore 53700, Pakistan; 2 Biotechnology Department, University of Malakand Chakdara Dir (lower), Khyber Pakhtunkhwa, Pakistan; 3 Zainab Memorial Hospital, Lahore, Pakistan

**Keywords:** HCV genotypes, Huh-7 cell line, Kaolinite

## Abstract

**Background:**

Hepatitis C virus (HCV) is estimated to infect 200 million individuals in the globe, including approximately 10 million in Pakistan causing both acute and chronic hepatitis. The standard treatment against HCV is pegylated interferon therapy in combination with a nucleoside analogue ribavirin. In addition, several herbal extracts and phytochemicals derivatives are used traditionally in the treatment of liver diseases as well as HCV infection. The present study determines the inhibitory effect of kaolin minerals compound against hepatitis C virus in Huh-7 cell lines**.**

**Methods:**

Huh-7 cell lines were used for the *in vitro* HCV replication by using HCV positive sera from different patients with known HCV genotypes and viral titer/load. Total RNA was extracted from these infected cells and was quantified by real-time polymerase chain reaction (Real-time PCR). The viral titer was compared with the control samples to determine the anti-HCV activity of kaolin derived compounds. Kaolin is a group of clay minerals, with the chemical composition Al_2_ Si_2_O_5_ (OH)_4_.

**Results:**

The results showed promising effectiveness of local kaolin derived anti-HCV compounds by causing 28% to 77% decrease in the HCV titer, when applied to infected Huh-7 cell lines. This study provides the basis for future work on these compounds especially to determine the specific pathway and mechanism for inhibitory action in the replicon systems of viral hepatitis.

**Conclusions:**

Kaolin mineral derivatives show promising inhibitory effects against HCV genotypes 3a and 1a infection, which suggests its possible use as complementary and alternative medicine for HCV viral infection.

## Background

Hepatitis C virus (HCV) is contributing increasing disease burden on global healthcare services and nearly 200 million people are infected worldwide by this virus [1,2]. Most of these infections cause chronic hepatitis and leading to progressive liver disease including cirrhosis, fibrosis and finally hepatocellular carcinoma (HCC) [3-5]. Fibrosis and cirrhosis is caused in up to 30% infected patients. It is estimated that 1-4% of the cirrhotic patients develop HCC [6-9]. Significant correlation of chronic HCV infection and HCC has been reported with genotype 3a in Pakistani population [10].

The current most effective therapy for HCV consists of pegylated interferon-α (IFN-α), administered once weekly plus daily oral ribavirin (RBV) for 24 to 48 weeks [11,12]. This combination therapy is quite successful in patients with HCV genotype 3 or 2 infection, leading to sustained virological response (SVR) in about 80-90% of patients treated. However, in patients infected with HCV genotype 1 or 4, only about a half of treated individuals achieve SVR [13,14]. Further, the treatment is often linked with severe side effects and high expenses. The HCV RNA level before therapy also effect treatment outcome in patients with genotype-1 infection. Patients with low baseline viral loads (e.g., <8 × 10^5^ IU/mL), have a better chance of SVR [15].

Beside the standard interferon plus ribavirin therapy, several plants, herb species and their derivatives have also been in use as potential anti-viral agents [16]. A number of active phytochemicals including the polyines, flavonoids, alkaloids, thiophenes, terpenoids and proteins have been identified to possess antiviral activity [17]. Most of these compounds have overlapping mechanisms of actions. Many medicinal plants are waiting to be exploited and evaluated for antiviral applications.

Kaolin is a group of clay minerals, with the chemical composition Al_2_ Si_2_O_5_(OH)_4_. It is a layered silicate mineral with one tetrahedral sheet linked through oxygen atoms to one octahedral sheet of alumina octahedral. It is also known as china clay or white clay. Mostly, white, brownish and grayish white in colour, also waxy and dull and contain common impurities like:Fe, Mg, Na, K,Ti, Ca, H2O [18]. Up to 350 mineral drugs have been identified and reported with medicinal value. Nearly 60 of these are still commonly used but prescribed alone rarely. However, they are usually given in combination with other components [19,20]. Now a days Selenium and its derivatives are favourable choice of complimentary alternative medicine (CAM) for liver diseases like HCV [21].

The main objective of the present study was to evaluated local kaolin mineral derivatives for their anti-HCV activity in the human hepatoma cell lines infected with HCV sera of different patients with known viral loads and genotypes.

## Methods

### Serum sample collection

Sera from more than 32 patients (12 and 20 samples of genotype 1a and 3a respectively) chronically infected with HCV infections was obtained from the molecular diagnostics facility, Centre for Applied Molecular Biology (CAMB), Lahore, Pakistan. The average serum viral loads of the samples were in the range of 227 × 10^3^ to 925 × 10^6^ IU/mL. All patients were negative for HBsAg. Informed written consent was obtained from the patients and the study was approved by the institutional ethics committee of National Centre of Excellence in Molecular Biology, Lahore.

### Cell lines

The Huh-7 and CHO cell lines were obtained from the Cell Bank of CEMB, University of the Punjab, Lahore.

### Kaolin derivatives

Total two different kaolin derivatives (kaolin minerals) were obtained in dimethyl sulfoxide (DMSO) as solvent, using modified method described by Alpert in 1984 [22]. The two different silicates minerals were obtained by using different soil from different places. Mineral derivative “A” as obtained from muddy soil while “B” was from sandy soil. Finally, these two compounds were selected for the determination of anti-HCV activity in *vitro* using Huh-7 cell lines. The mineral derivative of Kaolin was basically obtained from muddy and sandy soil.

### Physical properties of the kaolin

Mostly, White,Brownish white, Grayish white, Yellowish white and in color also Waxy, Dull and Pearly. Visible crystals extremely rare. Non fluorescent and Transparent to translucent diaphaneity etc.

### Chemical properties of the kaolin

Formula: Al_2_ Si_2_O_5_ (OH)_4_

Essential Elements: Al, H, O, Si

Common Impurities: Fe, Mg, Na, K, Ti, Ca, H_2_O

### Cell culture

The Huh-7 and Chinese hamster ovary (CHO) cell line were grown in Dulbecco’s modified eagle medium (DMEM) supplemented with 100 μg/ml penicillin, 100 μg/ml streptomycin and 10% fetal bovine serum (FBS) (Wisent INC., Montreal, Canada) incubated at 37°C with 5% CO_2_. They are highly permissive for the initiation of HCV replication.

### Cell splitting and plating

The Huh-7 cell line grown in DMEM supplemented with appropriate antibiotic, 10% FBS and CO2 was split and plated for toxicological analysis of kaolin derivatives.

### Anti-HCV analysis of kaolin minerals derivatives in Huh-7 cells

Huh-7 cell line was used for the *in vitro* HCV replication as described previously [23,24]. Total RNA was extracted from the Huh-7 cells infected with HCV serum using the Purescript RNA Isolation kit (Life Technologies, USA).

### HCV RNA quantitative detection in Huh-7 cells by real time PCR

HCV REAL-TM Quant SC kit (Sacace Biotechnologies, Italy) used in Smart Cycler (Cepheid) was used for Real-Time quantitative assay of HCV RNA in human plasma following procedure given in the kit protocol.

## Results

### Toxicity analysis of kaolin derivatives in Huh-7 cells

**Figure 1:**
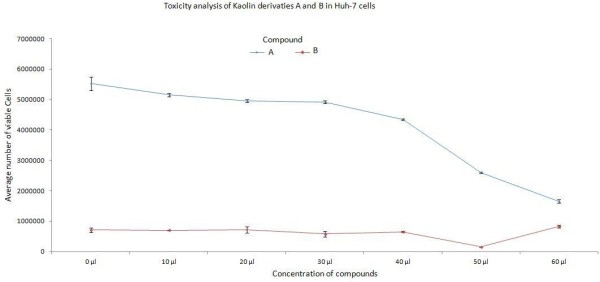
**Graphical presentation of toxicity analysis of kaolin derivatives A and B in Huh-7 cells treated at different concentrations from 10 - 60 μl.** Figure legend text.

Toxicity of kaolin derivatives was initially determined in Huh-7 Cell Lines. These cells were treated with different concentrations of the kaolin derivatives. After 24 hrs, cells were trypsinized and counted through haemocytometer. The viability of Huh-7 cells was confirmed at different concentrations by using trypan blue exclusion test. The concentration at which more than 70-80 percent of the cells survived was considered as non-toxic. 1X-PBS was used as a control. Administrations of up to 40 μl (50 μg/ml) of the local drugs (A and B) were found non-toxic to the cells (Figure 1).

### Antiviral analysis of kaolin derivatives in Huh-7 cell line

**Figure 2:**
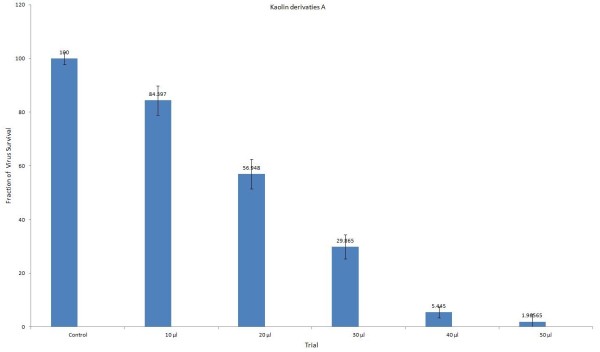
**Antiviral activity of kaolin derivatives A; Infected Huh-7 cells were treated with 10 to 50 μl concentrations for 24 hours.** HCV RNA levels are shown as a percentage relative to the levels of HCV RNA in cells incubated with control.

**Figure 3:**
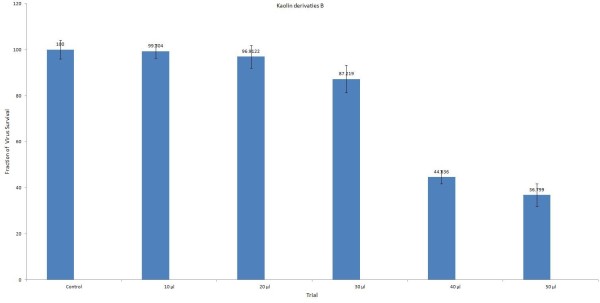
Antiviral activity of kaolin derivatives B; Infected Huh-7 cells were treated with 10 to 50 μl concentrations for 24 hours.

**Figure 4:**
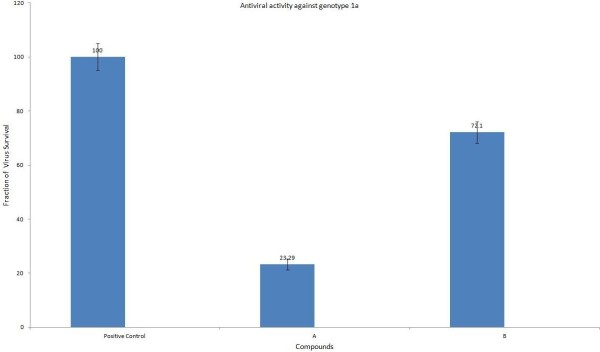
Antiviral activity of kaolin derivatives A and B; Infected Huh-7 cells were treated with 30 μl concentrations for 24 hours.

Antiviral activity of kaolin minerals derivatives “A and B” was determined in Huh-7 cells at different concentrations ranging from 10 to 50 μl (at concentration of 50 μg of kaolin derivatives/ml) as shown in the following Figures 2, 3 and 4. Both the local drugs were found non-toxic to the liver cells up to the administration of 30 μl dose. DMSO was used as a negative control while HCV infected sera was used as a positive control.

Viral titer of genotype 3a in infected Huh-7 cell lines was decreased up to 70% by using minerals derivatives “A” and 56% by administration of “B” as compared to the positive control samples. Similarly, reduction in HCV RNA of genotype 1a was observed up to 77% and 28% by administering “A and B” respectively. These results show that kaolin derivatives “A” is relatively more effective against genotype 3a and 1a, which suggests its use as complementary and alternative medicine for the treatment of HCV infection.

## Discussion

Currently, there is no antiviral drug completely effective against HCV infection [25]. The standard treatment against HCV infection is interferon therapy in combination with a nucleoside analogue ribavirin [13]. However, the treatment with pegylated interferon (IFN) plus ribavirin leads to a sustained virologic response in about 50% of patients [26] and lead to emergence of resistant quasispecies/strains due to increased drug pressure [27]. For rest of 50% non-respondents, supportive therapy with non-traditional compounds such as kaolin mineral obtained from the soil of different sources, which are suggested to have anti-HCV properties.

The present study describes antiviral activity of two conventional derivatives of kaolin mineral compounds against HCV in two different cell lines (*i.e.* Huh-7 & CHO cell lines). Different solvents such as dimethyl sulfoxide (DMSO), ethanol, methanol, acetone and water were used to determine solubility of the compounds. Huh-7 cell lines are important in studying antiviral activity *in vitro* as it allows HCV replication due to its expression of LDL-receptor molecules [28]. HCV genotype 3a and 1a were used for evaluation of antiviral responses. Viral titer was considered as an indicator of antiviral response. The concentrations of 50 μg/mL to 50 mg/mL with volume of 10-60 μl of both drugs were used.

Initially, different concentrations of kaolin derivatives “A and B” were analyzed for cytotoxicity in the Huh-7 and CHO cell lines. Both the compounds were found non-toxic up to the administration of 30 μl (50 μg of kaolin derivative/ml). At these concentrations cells did not display any significant cytotoxicity during regular cell passage and trypan blue counting. Cells viability was counted by haemocytometer and trypan blue stain method. The present study provides experimental data of local kaolin derived drugs for clinical treatment of chronic hepatitis C virus infection.

## Conclusions

We suggest that there could be excellent therapeutic agents in kaolin mineral compounds for the treatment of HCV infections. However, further studies are required to identify the specific active ingredients by using various techniques. This study also provides basis for future work on these compounds especially minerals derivatives A to determine its mechanism of action in the replicon systems and clinical trials for the treatment of the hepatitis C. Moreover, this study will be helpful to explore the new horizons of the discoveries of the kaolin derivatives, which shows promising inhibitory effects against HCV.

## Competing interests

The authors declare that they have no competing interests.

## Authors’ contributions

MI and LA conceived the study. LA conducted the experiments and analyzed the results. LA drafted the manuscript and made substantial contributions to design of the study. MI, MA, IR and AH critically reviewed the manuscript. AA, SAI and EHK participated in data analysis. All the authors studied and approved the final manuscript.
